# An enhanced procedure for urban mobile methane leak detection

**DOI:** 10.1016/j.heliyon.2020.e04876

**Published:** 2020-10-09

**Authors:** Tim Keyes, Gale Ridge, Martha Klein, Nathan Phillips, Robert Ackley, Yufeng Yang

**Affiliations:** a Evergreen Business Analytics, LLC, U.S.A.; b Steering Committee, 350 CT, U.S.A.; c Sierra Club Connecticut, U.S.A.; d Boston University, Department of Earth and Environment, U.S.A.; e Gas Safety, Inc., U.S.A.; f Institut National des Sciences Appliquées (INSA), Lyon, France

**Keywords:** Statistics, Environmental analysis, Environmental health, Environmental impact assessment, Waste, Public health, Methane leak detection, Cavity ring down spectroscopy, Geographic positioning system, Statistical analysis

## Abstract

Leaked methane from natural gas distribution pipelines is a significant human and environmental health problem in urban areas. To assess this risk, urban mobile methane leak surveys were conducted, using innovative methodology, on the streets of Hartford, Danbury, and New London, Connecticut, in March 2019. The Hartford survey was done to determine if results from a 2016 survey (Keyes et al., 2019) were persistent, and surveys in additional towns were done to determine if similar findings could be made using an identical approach. Results show that Hartford continues to be problematic, with approximately 3.4 leaks per road mile observed in 2016 and 4.3 leaks per mile estimated in 2019, similar to that previously found in Boston, Massachusetts (Phillips et al., 2013). A preliminary estimate of methane leaks in Hartford is 0.86 metric tonnes per day (or 313 metric tonnes per year), equivalent to 42,840 cubic feet per day of natural gas, and a daily gas consumption of approximately 214 U.S. households. Moreover, the surveys and analyses done for Danbury and New London also reveal problematic leaks, particularly for Danbury with an estimated 3.6 leaks per mile. Although road miles covered in New London were more limited, the survey revealed leak-prone areas, albeit with a range of methane readings lower than those in Hartford and Danbury. Data collection methods for all studies is first reported here and are readily transferable to similar urban settings. This work demonstrates the actionable value that can be gained from data-driven evaluations of urban pipeline performance, and if supplemented with a map of leak-prone pipe geo-location, and information on pipeline operating pressures, will provide a spatial database facilitating proactive repair and replacement of leak-prone urban pipes, a considerable improvement compared to reactive mitigation of human-reported leaks. While this work pertains to the selected urban towns in the Northeast, it exemplifies issues and opportunities nationwide in the United States.

## 1 Introduction

The objective of this paper is to introduce an innovative mobile methane leak detection method and to demonstrate its use in recent urban surveys. These survey results may be used to influence applicable legislation or policy changes to improve the manner in which gas companies in Connecticut and elsewhere manage gas pipelines and how they serve ratepaying customers. Most significantly, this research can be a guide for regulators to directly mitigate the effect of methane on public health and the environment. A related goal is to highlight the relatively efficient, effective, and pro-active manner in which surveys like the ones described in this report can be done by the Public Utilities Regulatory Authority (PURA) or the Local Distribution Companies (LDCs) in Connecticut, and their counterparts in other urban areas.

Natural gas is composed of 97% methane (CH_4_). Methane is a short-term pollutant with a half-life of seven years and has a much worse greenhouse gas impact than carbon dioxide (Intergovernmental Panel on Climate Change [IPCC], 2014). When measured over the life of the chemical, methane has as much as 100 times the climate impact of carbon dioxide. The Environmental Protection Agency (EPA) and Connecticut's Department of Energy and Environmental Protection (DEEP) both measure methane's climate harmfulness over 100 years which produces an artificially low Global Warming Potential (GWP) of 25 (Howarth, 2014). The high rate of unintentional leaks from hydraulic fracturing (fracking), gas pipelines, compressor stations, and other gas infrastructure, as well as the practice of intentional leaking or 'venting', contribute to methane's climate impact (Howarth, 2014). In addition to the severe climate impact, methane also kills trees, harms air quality, is an explosion hazard as has been reported in newspaper articles (Korte, 2018; Santora, 2014), and increases the risk of pediatric asthma in children living in homes that are connected to natural gas for heating and/or cooking as reported by the non-profit Home Energy Efficiency Team of Massachusetts [HEETMA] (Krasner and Jones, 2019).

While the greenhouse gas impact of methane is important, of more immediate concern for most residents is the negative public health impacts that are caused by leaking methane. Natural gas that flows through Connecticut pipelines is fracked from the Marcellus Shale in Pennsylvania and Ohio and includes other components in addition to methane. Some of these are volatile organic compounds which lead to the formation of ground level ozone smog that exacerbate asthma and emphysema, impairing lung function and other pre-existing respiratory problems: benzene, which is linked to cancer, respiratory illnesses, and birth defects; ethylbenzene, linked to neurological and blood disorders; and formaldehyde, which is linked to certain cancers and respiratory illnesses (Pun et al., 2015; Shi et al., 2016). Fracking generates at least two thirds of natural gas distributed across the United States (U.S. Energy Information Administration, 2016a, b).

The volume of natural gas leaked in cities and the concomitant climate and economic impacts can be substantial. A recent study in Boston (McKain et al., 2015) estimated a gas loss rate from greater Boston at 2.7% of the total consumed natural gas, which resulted in an annual value loss of approximately $90 million, partially borne by ratepayers. This represents approximately 10% of Massachusetts' greenhouse gas emissions inventory. Estimates of tree damage from gas leaks in mid-sized municipalities in Massachusetts range from hundreds of thousands to millions of dollars. Urban natural gas pipelines are mostly made up of ‘distribution’ pipelines operating at low pressures (below 100 pounds per square inch [psi]) compared to the high-pressure interstate transmission pipelines that deliver natural gas into high population areas and the “upstream” pipelines in production areas. In older cities such as those in the eastern U.S., leak-prone cast iron, wrought iron, or bare steel gas pipelines can be up to a century old or more. These pipes often leak at decaying joint connections (typically spaced at 12-foot intervals), or due to corrosion or mechanical disturbance caused by freezing conditions. While an inventory of leak-prone pipes for Hartford was unavailable for this study, it is known that Connecticut has some of the highest total miles and percentage of leak-prone pipe in the U.S. (Pipeline Hazardous Materials and Safety Administration, 2015–17). In general, independent research using precise measuring devices, such as the Picarro Cavity Ring-Down Spectrometer used in this study, documents more gas leaks than are reported by state regulatory agencies (see Figure 4 below; also, Home Energy Efficiency Team (HEET), n.d., in references). Studies done in Weston, Acton, and Fitchburg, Massachusetts, in 2019, 2017, and 2014, respectively by the authors (Gas Safety, Inc.) illustrate this point. In Weston, 292 leaks were found, compared with 117 reported; in Acton, 234 vs. 115; in Fitchburg, 177 vs. 42. Data reported to regulatory agencies are available via FOIA request, and are tracked at the HEET site (HEET, n.d.). Gas Safety data are available upon request to the authors.

**Figure 1:**
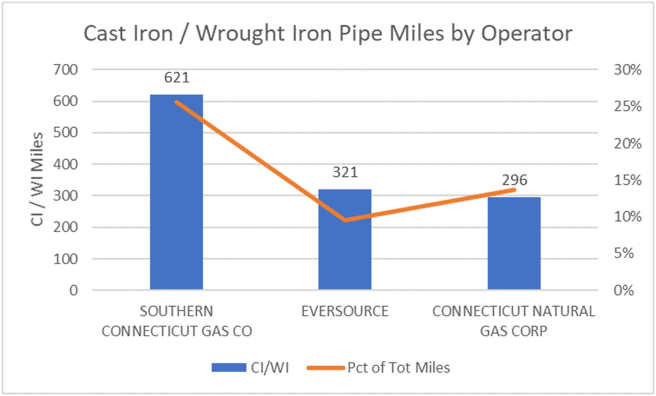
Mileage of cast iron pipe managed by Connecticut operators.

There are three investor-owned natural gas companies (LDCs) in Connecticut serving urban and suburban communities. These are Connecticut Natural Gas (CNG; cngcorp.com), Southern Connecticut Gas (SCG; www.soconngas.com), both owned by UI/Iberdola, and Eversource/Yankee Gas Company (ES; www.eversource.com). Gas service in Hartford is supplied by CNG, the smallest of the three main gas delivery companies in terms of miles of pipeline mains (with 296 miles of leak-prone cast iron and wrought iron mains in 2017 (14% of their total miles), according to the Pipeline Hazardous Materials Safety Administration (PHMSA, 2015–17)). Hartford is the largest city within the CNG service area. Gas service in Danbury and New London is supplied by ES. See Figure 1.

There were 225.45 road miles in Hartford as of December 31, 2014 (Connecticut Department of Transportation, 2014). Strictly speaking, this study detected CH_4_ leaks as a broader category than natural gas leaks. Methane can originate from sources other than natural gas pipelines, including broken sewer mains, landfills, and wetlands. Prior work in Boston (Phillips et al., 2013) showed that the vast majority of leaks detected from under streets and sidewalks bore a distinct chemical signature of natural gas methane. Moreover, the spatial signature of wetland and landfill leaks is distinctly different from that of pipeline leaks. Pipe leaks are recognizable as abrupt and highly localized spikes in methane concentration, while wetland and landfill methane emissions are more diffuse gradual deviations from a baseline methane concentration. Emissions of CH_4_ from sewer systems indicate a “non-flowing” or “dead spot” in the system and are typically rare occurrences, but there is no guarantee each leak indication detected in this study was from a methane pipe. While confirming that each leak is from a methane pipe is beyond the scope of this study, the leak indications found were too numerous to have significant representation from sewer pipelines, wetlands or landfills.

Surveys were conducted mid-February through late March 2016 in Hartford, and again in mid-late March in 2019 in Hartford, Danbury, and New London, CT, a timing that suppressed alternative methane emission signals from possible wetland, landfill or other subsurface sources, because of cold temperatures. Danbury and New London were selected for surveys owing to a similar aging infrastructure, and in order to test the hypothesis that similar leak-prone pipeline data would be observed. The 2016 survey in Hartford was nearly a complete census of all 225 road miles in the town (Connecticut Department of Transportation, 2014), whereas the 2019 surveys done in Danbury and New London were samples of road miles in each town, targeting areas in which pipelines are most likely to be present, based on the judgment of the field team. As a result, comparing results from Hartford across time requires recognition that the 2019 survey was a smaller sensor sample (not a census of road miles). Additionally, for comparison, data collected from the survey was placed precisely between the first and last dates of a PURA one-year reporting period (October 15, 2015–October 15, 2016).

## 2 Materials and methods

The same mobile Picarro G2301 Cavity Ring-Down Spectrometer (Picarro, Inc., Santa Clara, CA; http://www.picarro.com/) was used in all surveys (2016 and 2019), installed in a vehicle equipped with a geographic positioning system (GPS), and driven on the public roads in each town. Focus on the 2019 Hartford survey was on main roadway arteries and problematic areas based on 2016 conclusions. A filtered inlet tube was placed outside the passenger side of the vehicle. The analyzer was periodically tested with 0 and 5 ppm CH_4_ test gas (Spec Air Specialty Gases, Auburn, ME; www.mainespecialtygases.com/; reported precision ±10%) throughout the 2016 survey.

As roadways in the town being surveyed are driven, the system records parts per million (ppm) CH_4_ concentration each second, along with latitude-longitude GPS coordinates. The system operator will typically start and stop the recording of data into individual files representing survey micro-areas likely to have similar ambient conditions, and therefore each town survey results in many individual files of CH_4_ readings by geo-position. For example, the 2016 survey in Hartford produced 65 data files.

To distinguish discrete leaks from the spatially continuous raw methane concentration data, a modified Tau approach (Olewuezi et al., 2015) was used to perform outlier detection on the raw spatial methane concentration data. This method is a statistical approach to support deciding whether to keep or discard suspected outliers in a population sample, in this case an individual CH_4_ system file representing a micro-area within the town being surveyed. A threshold methane level that meets the outlier category, indicating a leak, is calculated by the data file's CH_4_ sample size, sample average, sample standard deviation, and desired confidence level.

To avoid double-counting leaks that were driven past multiple times, a procedure was used to eliminate multiple outliers within a spatial window of 30 m radius from the highest peak methane concentration in the vicinity. A spatial window was used from as small as 5 m up to 30 m. It was found that there was relative insensitivity of the total leak count in this range, while apparent leak count decreased substantially in window sizes above 30 m. Since vehicle lane widths are generally approximately 10 m or less, the 30-meter window is large enough to prevent double-counting but small enough to avoid incorrectly combining separate observed leaks into one.

Once each town's survey was complete, the corresponding data files described above were created (.DAT and.KML) for use in subsequent analysis, the essential steps of which are outlined in the Appendix, which also contains the file control lists, software (R code) for processing the data files, and the resulting outlier files (predicted leaks).

Over the last six years a number of mobile methane leak mapping studies have been conducted (Chamberlain et al., 2016; Gallagher et al., 2015; Hopkins et al., 2016; Jackson et al., 2014; Phillips et al., 2013; etc.) All of these studies contain elements of arbitrary, or geographically idiosyncratic, spatial, temporal, or scalar concentration threshold in which continuous CH_4_ readings are discretized into leaks as identifiable objects. Table 1 displays a selection of recent studies attempting to define discrete CH_4_ leaks from mobile CH_4_ mapping data.

**Table 1:** A selection of relevant recent studies.

Year	Study	Definition of leak	Notes
2013	Phillips et al.	“...a unique, spatially contiguous group of [CH_4_] observations, all values of which exceed a concentration threshold of 2.50 ppm...correspond[ing] to the 90th percentile of the distribution of data from all road miles driven, and, relative to global background, is 37% above 2011 mean mixing ratios observed at Mauna Loa…”	
2014	Jackson et al.	“...a separate, spatially contiguous set of [CH4] observations exceeding a concentration threshold of 2.50 ppm at >5-m spacing.”	
2015	Gallagher et al.	“... a spatially contiguous set of [CH_4_] observations greater than 2.5 ppm (i.e., >20% above background [CH_4_] of 1.8–2.0 ppm of CH_4_) with a distance threshold radius greater than 5 m from any other elevated [CH_4_] observation.”	
2015	Lamb et al.	“Specific pipeline leaks and facilities were selected randomly from Local Distribution Company leak survey data and facility lists for the targeted areas.”	Leaks not defined in study; obtained elsewhere
2016	Chamberlain et al.	“...temporally discrete CH_4_ concentration peaks that were above the 98% percentile of all measured concentrations (>1.93 ppm). Survey time series were de-trended prior to peak identification to control for fluctuations in background CH_4_ concentration throughout the day.”	
2016	Hopkins et al.	“We defined hot spots as road segments where at least one 150 m segment had a CH4 value that exceeded the 95th percentile threshold (132–360 ppb above the local background level). The spatial extent of each hot spot was defined by the number of adjacent 150 m road segments that had CH4 values above the local background level. Local background CH4 levels varied over the course of each transect due to spatial variability and diurnal changes in boundary layer height and were thus determined by visual inspection...”	
2016	Lamb et al.	“These results were accumulated in 100 × 100 m grids and averaged to produce a map of average methane enhancement for the urban area. These gridded results were filtered to remove levels greater than 500 ppb to eliminate the presence of large, identified sources.”	
2017	Von Fischer et al.	“...as a set of elevated readings <160 m that was found more than once”. “...elevated” CH_4_ concentrations as >10% or >1SD above background, whichever is greater.”	
2019	Weller et al.	“...we define an elevated reading as any reading having CH_4_ levels greater than or equal to 110% of the baseline value. Because the baseline value will vary in time and space, so will the threshold for elevated CH_4_ levels, but at a typical background of 2 ppm, the threshold is 2.2 ppm”	Includes much additional algorithm detail not included here.
2020	This study	Uses modified Thompson's Tau method to identify outlier measurements relative to each local ambient level, dynamically determined.	

This study also contains a spatial element which is arbitrary, but we believe advances toward a goal of standardized gas leak identification by employing a statistical data grouping method that will have broad general utility.

## 3 Results from 2016 study

In the 2016 study, a total of 716 distinct methane leaks over 225.45 road miles in Hartford were detected, resulting in a leak frequency of 3.2 leaks per road mile. This leak frequency compares to 4.3 leaks per mile previously discovered in Boston, MA (Phillips et al., 2013). Over a one-year period covering the same area (15 October, 2015 to 15 October, 2016), PURA recorded138 leaks (data provided by PURA). When the researchers who performed the 2016 study compared the number of leaks found in a period of weeks to the number reported by gas companies that are recorded and monitored by PURA over an entire year, they objectively measured approximately five times more leaks than were recorded by PURA. The difference between the number of leaks identified by pro-active, objective measurement is significantly larger than those found by incidental finding or report. LDCs are required by PURA to record any leak reported by customers or employees into their data. Any hazardous leaks (e.g., Grade 1) are repaired immediately. Leaks that represent a potential future hazard (e.g., Grade 2) are periodically monitored and scheduled for repair. Grade 3 (the lowest risk category) leaks are monitored annually, but data are not available in detail.

A preliminary estimate of the leakage rate from the leaks found during the survey was made using the leak size distribution data from a prior Boston study (Hendrick et al., 2016). Assuming leaked natural gas volume from the Hartford pipes had the same distribution found in the Boston study, with a log-normal average of 1.2 kg CH_4_ per day, a preliminary estimate would find 0.86 metric tonnes/42,840 cubic feet of gas was lost to the atmosphere each day, resulting in 313 metric tonnes per year. Although peak methane concentrations observed from the mobile survey offered a rough indication of leak size, it is not a reliable indicator of this, because shifting wind speed and direction influences leaked gas concentrations from moment to moment. To mitigate this risk, we attempted to take measurements as close to the pipeline route as possible. What is important is to detect a deviation from the CH_4_ baseline that indicates a fugitive emission.

The 0.86 metric tonnes of methane loss per day in Hartford compares to the estimate of 4.0 metric tonnes of methane loss per day in in Boston. Note Boston has 3.5 times more public road miles than Hartford with 790 miles (Phillips et al., 2013). The Hartford leak rate represent an equivalent daily gas consumption of approximately 214 U.S. households. While this number is a small fraction of total households in Hartford, equal to approximately 45,800 (U.S. Census Bureau, 2018), the clear benefits to air quality, tree health, and public and private property safety of repairing leaks, as well as economic benefits should add impetus to the City of Hartford to address the problem.

From data provided to PHMSA (2015–17), in 2015 Connecticut ranked sixth among U.S. states in terms of the total number of miles of leak-prone, cast iron and wrought iron gas distribution pipeline, and first in miles of cast iron and wrought iron pipe in percentage of total miles (16.9%) of distribution pipeline. By comparison, Massachusetts ranked third and fourth in these categories, respectively. From 2005–15, according to the U.S. Energy Information Administration (U.S. Energy Information Administration (EIA), 2016a, b), CNG reduced its inventory of leak-prone cast iron and wrought iron pipe by 24%, about the same as the reduction attained by ES, and at twice the rate over the same period by the largest gas utility in the state, SCG (12% reduction from 2005–15).

**Figure 2:**
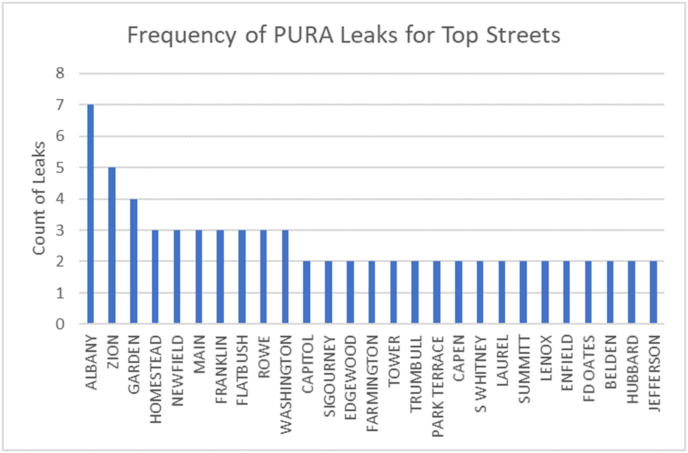
Frequency distribution by street name from leak reports recorded by PURA between October 15, 2015 and October 15, 2016, a one year, four season period. A total of 138 gas leaks reported in the city of Hartford.

**Figure 3:**
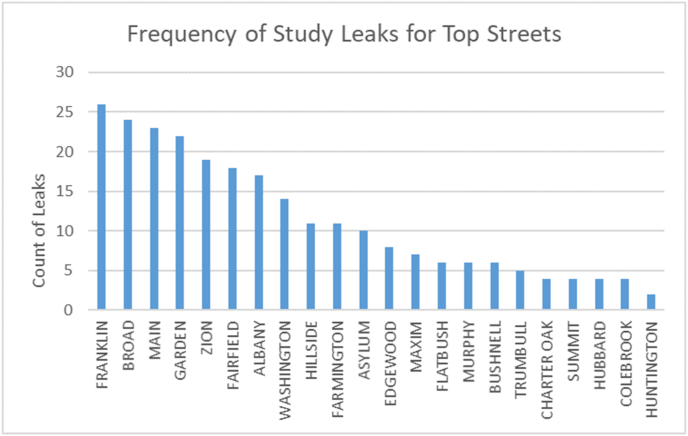
Frequency distribution by street name of Leaks from this study between February 25 through March 31, 2016. A total of 716 gas leaks were recorded in the city of Hartford.

The data from the two sources indicate that Hartford is substantially less prone to gas leaks than Boston, and may be in better shape than cities served by SCG. This may be attributed to better efforts by CNG to repair and replace leak-prone pipe in Hartford compared to National Grid, the gas utility serving most of Boston, or SCG, which has reduced its leak-prone pipe miles by half the rate of CNG during the last decade (PHMSA, 2015–17). Figures 2 and 3 show the frequency of leaks recorded by PURA and those found in this study, by street name. PURA and the study clearly differ in terms of count and location. For example, see leaks associated with Franklin Street.

In 2014 “An Act Concerning Lost and Unaccounted for Gas” (PA 14–152) was passed by the Connecticut State legislature. This two-paragraph act permits Connecticut natural gas distribution companies to charge Connecticut ratepayers fees not clearly identified in their bills for lost unused gas. These companies are permitted to estimate the volume of “lost and unaccounted for” natural gas escaping from their equipment and charge customers to recoup the revenue. This provides a disincentive for repair and/or replacement of faulty pipes and joints in a timely manner, because it rewards the companies for leaking rather than penalizing them. As written earlier, it is estimated the State of Massachusetts currently loses approximately $90 million/year in lost value of leaked gas to the atmosphere. Though lacking reliable data, it can be inferred that the Connecticut natural gas pipeline system is also losing millions of dollars per year of gas revenue to the atmosphere.

The State of Connecticut possesses an energy policy which is committed to expanding public and corporate use of natural gas. Additionally, it supports the expansion of large capacity high pressure Interstate transmission pipelines through Connecticut from frack gas providers to consumers, international exporters, and business entities in other regions of the U.S. and Canada. Pressure is maintained using compressor stations spaced at intervals along the transmission pipelines. Evidence in the literature strongly suggests a correlation between exposure to gas infrastructure such as compressor stations and negative health outcomes. Compressor stations produce pollutants such as volatile organic compounds and particulate matter, which can be human carcinogens (Russo and Carpenter, 2019), and in a perspective to the New England Journal of Medicine, was noted to cause increased rates of respiratory illness, cardiovascular disease, and premature birth (Landrigan et al., 2020). Compressor stations release billions of tons of greenhouse gases, and the amount of methane released can vary widely, at times exceeding EPA standards (Subramanian et al., 2015; Payne et al., 2017). Compressor stations produce noise pollution (Boyle et al., 2017) which can cause stress, sleep loss and cognitive deficits (Landrigan et al., 2020). Homes with gas stoves have elevated levels of certain pollutants such as fine particulate matter and nitrogen dioxide that at times exceeded EPA standards (Singer et al., 2017), and the risk of asthma in children is higher in homes with gas stoves (Lin et al., 2013).

PURA is responsible for intrastate gas pipeline safety oversight, and is authorized by PHMSA/Office of Pipeline Safety to monitor interstate pipeline safety. PURA claims it records and monitors all leaks that are reported to it. A concern is that ES, CNG, and SCG have been reported to check their pipes remotely and electronically which may result in underestimation of gas leak events.

Given the results from the study, the rate of methane leaks appears to be much higher than PURA records show, and the gas expansion plan should be re-evaluated in light of this. The solution to the problem is legislative, and there should be a zero-leak tolerance policy maintained for natural gas pipelines, as there is for petroleum pipelines (Clean Water Act 33 U.S.C. 1251 et seq.).

**Figure 4:**
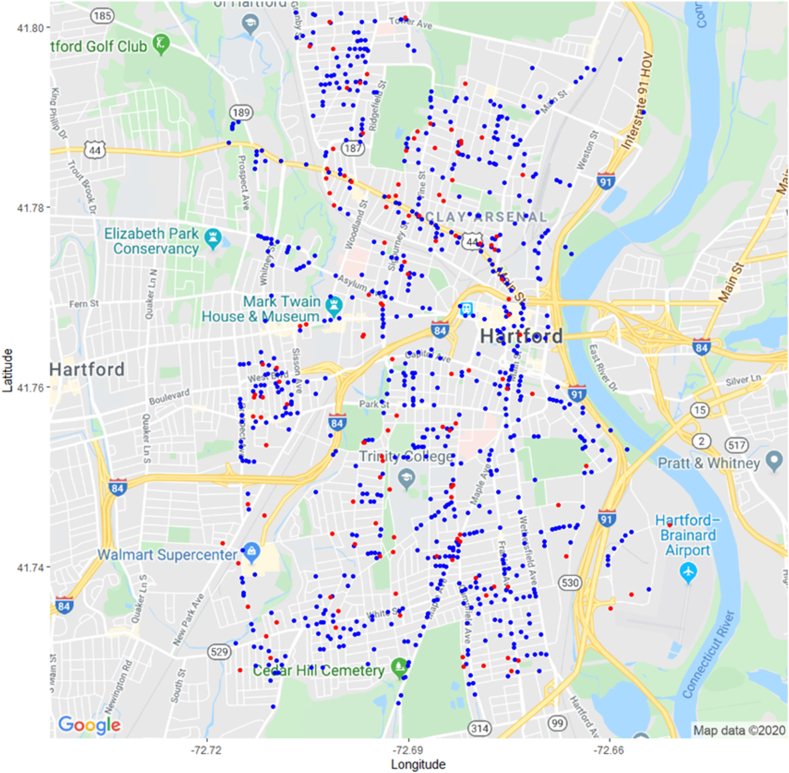
Locations of methane leaks found in Hartford; data plotted in blue (716 leaks) gathered in this study February 25 through March 31, 2016, and in red by PURA (138 leaks of Grade 1 and 2) October 15, 2015–October 15, 2016. Details for Grade 3 leaks not available to authors.

**Figure 5:**
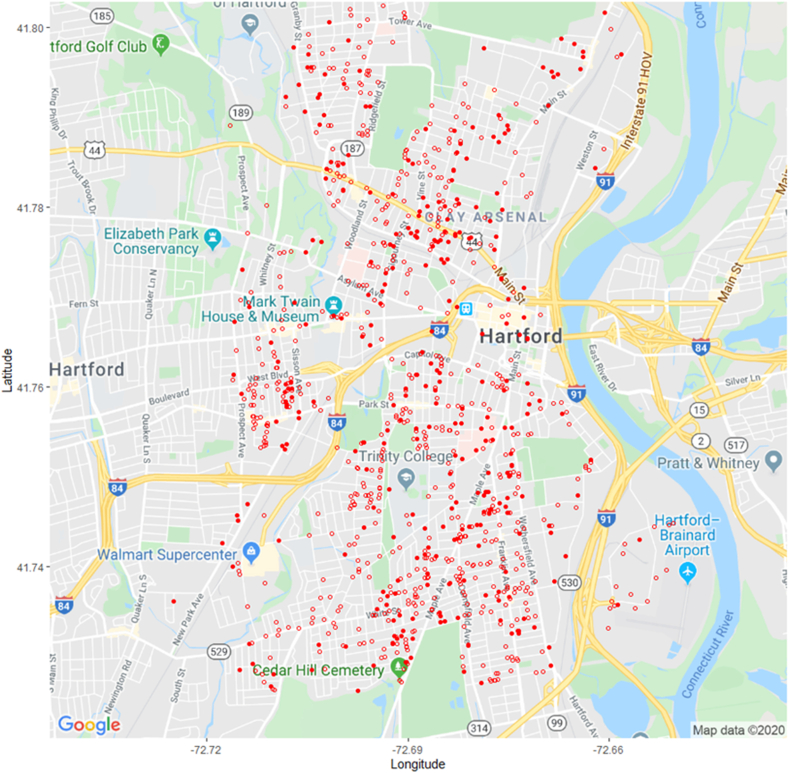
Locations of PURA Methane Leaks Found in Hartford, 2011–2016 (1,069 leaks, 321 of Grade 1 and 748 of Grade 2; Grade 1 leaks are red filled circles, Grade 2 are not filled). Details for Grade 3 leaks not available to authors.

To recap, PURA categorizes gas leaks into three classes or grades; Grade 1 (existing hazardous leak), Grade 2 (potential future hazardous leak), and Grade 3 (non-hazardous at the time of detection, expected to remain non-hazardous, and not required to be repaired). Grades 1 and 2 are reported to PURA in detail. Additionally, PURA does not require the volume of natural gas loss in Grades 1 and 2 to be reported. Grade 3 leaks are often numerous and may be seen as problematic, because a) they may progress into a higher Grade (i.e., are misclassified), b) the accumulated number of small leaks become equal in volume loss to fewer higher volume leaks and c), the often more widely distributed Grade 3 leaks may cause more human and environmental harm over a wider geographic area. Figures 4 and 5 plot the location of leaks found in this study, and leaks reported to PURA. Note that graphical representation of each leak is of constant size in Figure 4, as there was no direct method of comparison of the authors' study to PURA's.

From evidence provided in this study, it may be suggested that a proactive alternative to the PURA model of chiefly reactive reporting may be both economically and environmentally superior. This study identified natural gas leaks using vehicle mounted sensing equipment, while the PURA model appears to largely be dependent on voluntary human reporting. Considering the relative ease with which testing equipment can be mobilized, a proactive approach may be advantageous in terms of public health, the environment, and long-term cost-benefits to these urban areas, the state, and gas companies as well. The study illustrates a means to improve gas industry system-wide performance as well as enhance public understanding of the efficacy of mobile gas leak street-level monitoring.

## 4 Results from 2019 study

The 2019 study commenced with a revisit and update to the 2016 study done in Hartford, then expanded to other Connecticut towns. The leak-detection algorithm is identical between the two periods, with an updated approach to de-duplicating neighboring outliers, using the 30-meter spatial window, for final leak determination.

### 4.1 Town of Hartford

#### 4.1.1 Updated 2016 survey results

**Figure 6:**
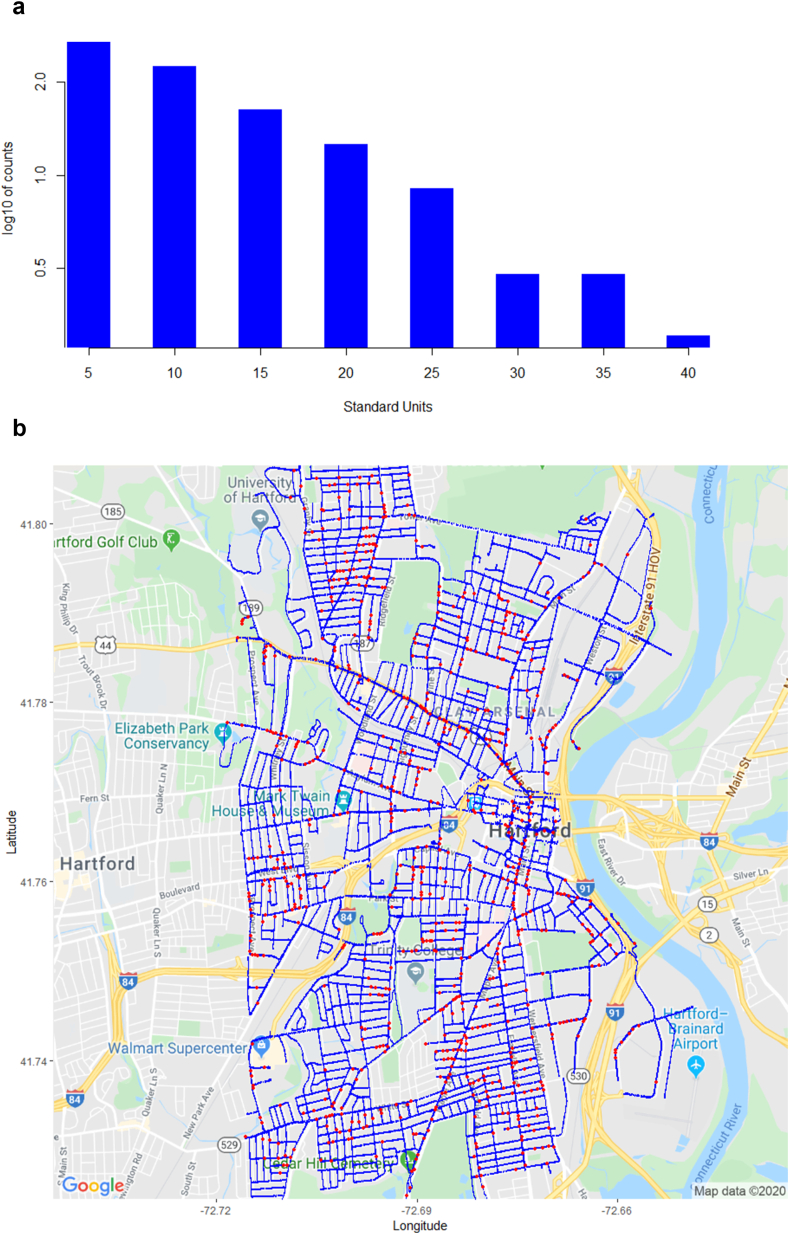
a: (log10) Histogram of z-scores for Predicted Leaks in Hartford, 2016. b: Updated 2016 Hartford Survey Results Map (blue = measurement, red = leak).

The revisited survey resulted in 766 leak detections found (as compared with 716 originally). Table 2 below displays key results from the Hartford, 2016 mobile methane survey. Further work on this survey was reported in Keyes et al. (2019). Figure 6a displays a histogram of standardized outliers, or “z-scores,” assessing the statistical distance in standard deviation units that an outlier measurement is away from its local ambient mean level of methane. Note that counts are on the log10 scale. Clearly from this graph there are methane leaks significantly higher than ambient levels.

**Table 2:** 2016 Hartford survey results.

Survey	Measurements	Road Miles	Leaks	Leaks/Mile	Min CH_4_ (ppm)	Max CH_4_ (ppm)
Hartford, 2016	140,602	225	766	3.4	1.93	9.67

Figure 6b below shows a map of recorded measurements in blue, and predicted leaks in red.

#### 4.1.2 2019 survey results

**Figure 7:**
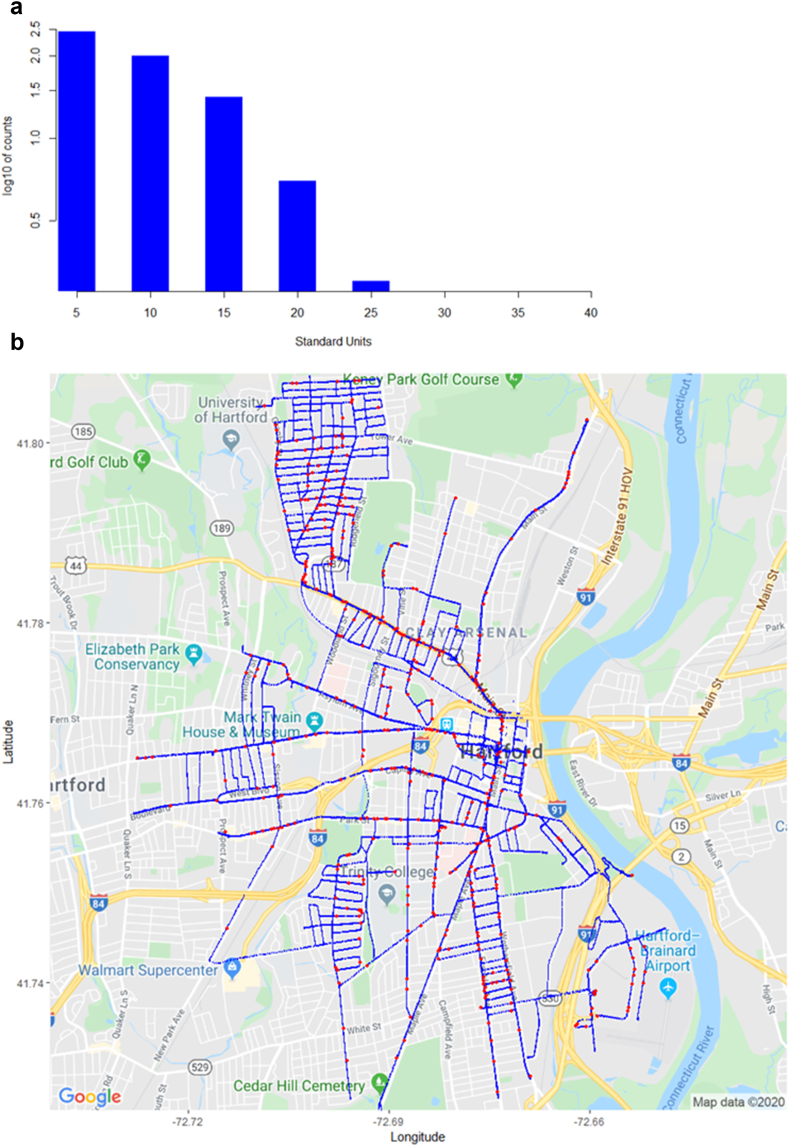
a: (log10) Histogram of z-scores for Predicted Leaks in Hartford, 2019. b: 2019 Hartford Survey Results Map (blue = measurement, red = leak).

Table 3 below displays key results from the Hartford mobile methane survey done in 2019. Figure 7b illustrates that potential leaks continue to be distributed throughout the town. Given that this survey was not a census (i.e., not all 225 road miles of Hartford were driven), the Road Miles entry is a figure estimated using a ratio of the number of measurements taken in the 2019 study (62,546) to the number of measurements taken in the 2016 study (i.e., 100 miles ~62,546/140,602 × 225 miles). In our methodology, both sides of each roadway are driven, occasionally more than once, to isolate problematic areas in terms of CH_4_ readings. We have purposively de-duplicated peak methane measurements in our algorithm, using spatial coordinates, but have yet to formulate a more precise determination of road miles driven (or the length of all roads on which measurements have been taken), which we leave for future surveys. Assuming reasonably that the surveying vehicle is travelling at the same speed in each survey, and each side of each road is surveyed, then a constant one-second sensor refresh rate implies that the total number of sensor readings is indicative of total miles driven. Note both the min and max leaks recorded in 2019 are above similar measurements from 2016, indicating that leak-prone pipelines in Hartford continue to be problematic, if not worsening as compared to 2016's survey. Figure 7a displays a histogram of standardized outliers. Owing to the log10 scale, singleton outliers in the 35 + range are plotted at zero.

**Table 3:** 2019 Hartford survey results.

Survey	Measurements	Road Miles	Leaks	Leaks/Mile	Min CH_4_ (ppm)	Max CH_4_ (ppm)
Hartford, 2019	62,546	100	425	4.3	1.97	10.99

Figure 7b below shows a map of recorded measurements in blue, and predicted leaks in red.

#### 4.1.3 Comparison of 2016 and 2019, and conclusions

Figures 6a,b and 7a,b suggest that leaks throughout the town continue to be problematic, and are persistent, for example along Albany Avenue/Main Street, Maple Avenue and the Keney Park area to the north of town, and east of the University of Hartford. Tables 2 and 3 indicate that the estimated number of leaks per road mile has increased from 3.2 per mile, to 4.3 per mile (coincidentally similar to the figure resulting from the earlier Boston study), although the latter figure is based on an estimate of road miles driven.

### 4.2 Town of Danbury

**Figure 8:**
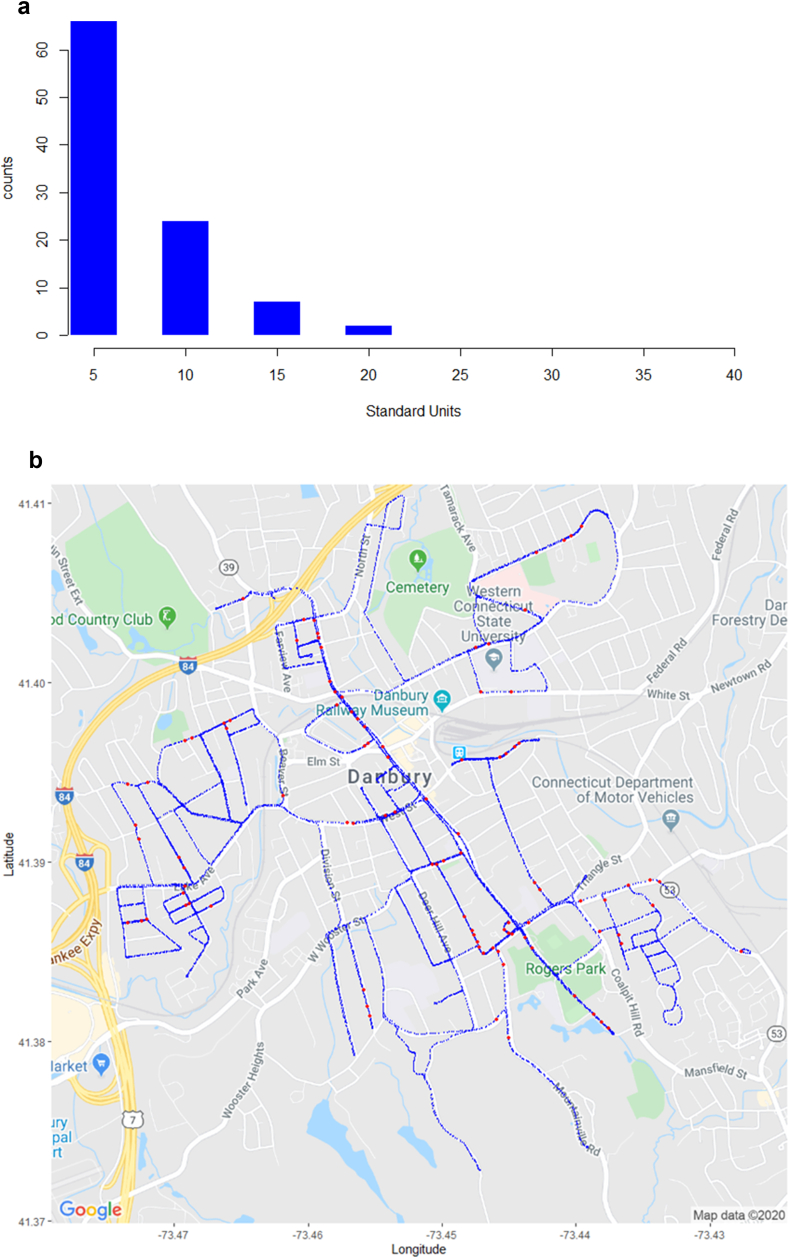
a: Histogram of z-scores for Predicted Leaks in Danbury, 2019. b: 2019 Danbury Survey Results Map (blue = measurement, red = leak).

Table 4 below displays key results from the 2019 mobile methane survey for Danbury. Again, an estimate is made for the road miles driven, using the methodology explained above. Figure 8a below displays the histogram of standardized outliers, no longer on the log10 scale, and Figure 8b shows a map of recorded measurements in blue, and predicted leaks in red. This evidence shows there is a cluster of predicted leaks in the vicinity of Rogers Park, and also near the Railway Museum. The estimated leaks per mile is 3.6, consistent with the 2016 and 2019 results for Hartford.

**Table 4:** 2019 Danbury survey results.

Survey	Measurements	Road Miles	Leaks	Leaks/Mile	Min CH_4_ (ppm)	Max CH_4_ (ppm)
Danbury, 2019	17,120	27.4	99	3.6	1.99	5.15

### 4.3 Town of New London

**Figure 9:**
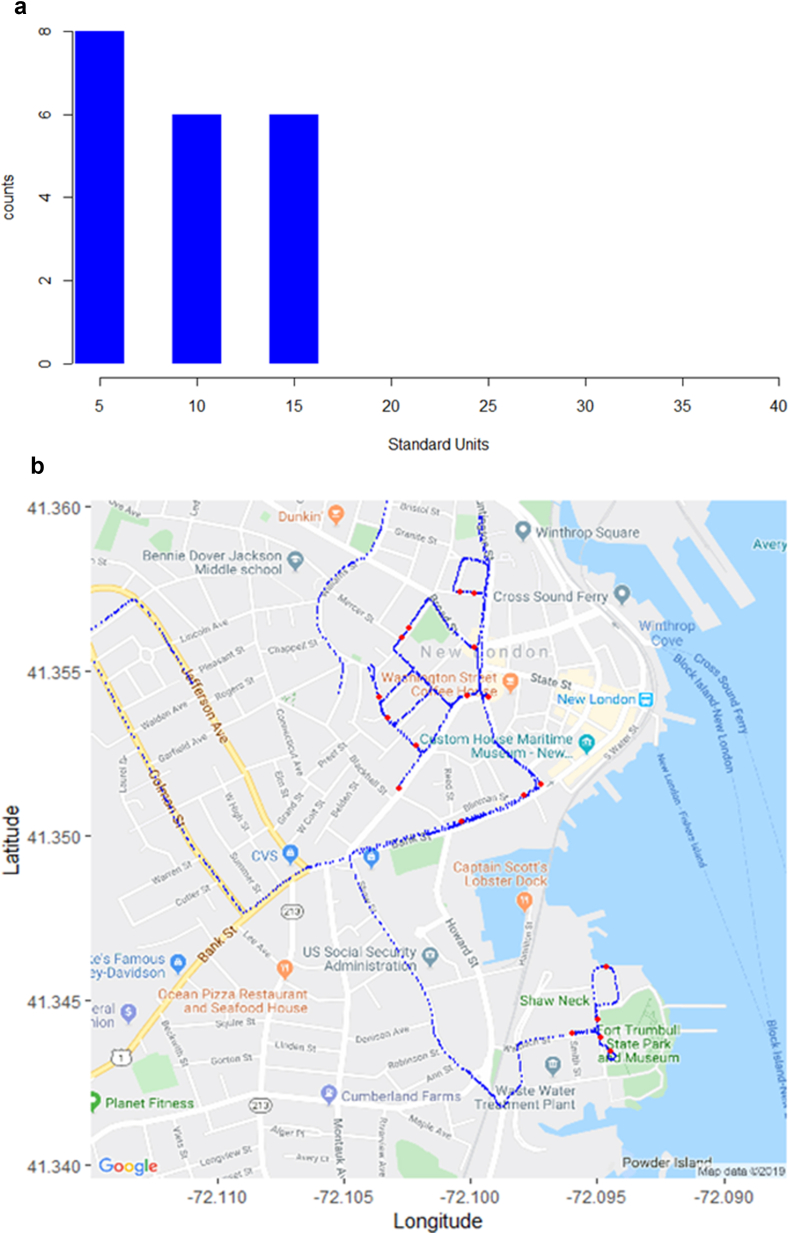
a: Histogram of z-scores for Predicted Leaks in New London, 2019. b: 2019 New London Survey Results Map (blue = measurement, red = leak).

Table 5 below displays key results from the 2019 mobile methane survey. Given the relative short distance covered (based on the estimation approach), the Leaks/Mile figure should be used with caution. Figure 9a shows the histogram of standardized outliers, not on the log10 scale. From Figure 9b, most of the leaks in New London appear to be in the central part of the town and in the Fort Trumbull area. The range of CH_4_ ppm is relatively lower than that of Hartford and Danbury.

**Table 5:** 2019 New London survey results.

Survey	Measurements	Road Miles	Leaks	Leaks/Mile	Min CH_4_ (ppm)	Max CH_4_ (ppm)
New London, 2019	5,096	7.6	20	2.6	2.00	2.59

In conclusion, this report reflects the results of 2019 mobile methane surveys conducted in Hartford, Danbury, and New London, CT, with the Hartford results compared with a similarly executed survey done in 2016. The results support that methane leaks remain prevalent and persistent in Hartford and are also present in other Connecticut towns; Danbury has a leak propensity similar to that in Hartford, while New London has problematic leak indications, but less pronounced CH_4_ readings than those in Hartford and Danbury.

This study outlines and demonstrates a straightforward yet innovative approach to pro-active leak management – one that could be employed by regulators or LDCs to improve system performance, or by state legislators to evaluate energy management policies.

Further advances to the analytic methodologies may include overlaying pipeline (methane and sewer) grid locations and methane pipeline operating pressures with the predicted leaks identified as a result of a survey, with the aim of further verifying (beyond the validation work done in 2016), that leaks can be assigned to specific pipeline sections, and therefore to specific remediation actions. These data may also help explain why certain roadways in surveyed towns possess a higher spatial density of leaks than others and would allow for an estimate of the likely rankings of leak rates from particular lengths of pipeline. Among the low-pressure distribution pipelines, operating pressures can vary substantially, from 0.5 psi to 60 psi or more. A pipe will leak at a rate that is proportional to the pipeline operating pressure, so leaks found in zones of higher operating pressure will be expected to leak higher volumes of natural gas. Proactive measures and management of all gas pipeline system defects, from small to large, with transparency taking into account both the frequency and severity of leaks, using established risk management procedures is recommended.

**Key findings**

1. Leaked methane from natural gas distribution is a significant human and environmental health problem in urban areas.
2. A straightforward, innovative, and proactive measurement procedure is introduced and deployed in towns in the Northeast U.S. The procedure is easily transferable to any similar urban setting and is superior to current regulator practices.

## Declarations

### Author contribution statement

Tim Keyes: Conceived and designed the experiments; Performed the experiments; Analyzed and interpreted the data; Contributed reagents, materials, analysis tools or data; Wrote the paper.

Gale Ridge, Martha Klein, Nathan Phillips: Conceived and designed the experiments; Analyzed and interpreted the data; Wrote the paper.

Robert Ackley: Conceived and designed the experiments; Performed the experiments; Analyzed and interpreted the data; Wrote the paper.

Yufeng Yang: Conceived and designed the experiments; Analyzed and interpreted the data; Contributed reagents, materials, analysis tools or data; Wrote the paper.

### Funding statement

This work was supported by the Sierra Club.

### Competing interest statement

The authors declare the following conflict of interests: Tim Keyes and Robert Ackley were paid consultants, commissioned by the Sierra Club Connecticut for this work.

### Additional information

Data associated with this study has been deposited on Google Drive at the following locations: https://drive.google.com/drive/folders/17KOqmlky6Tx_aIOlYi0v2TBRBuZFepkz; https://drive.google.com/drive/folders/18oCTzG0Jp8jTDP9y8btJvAFbJvDLvAMh; https://drive.google.com/drive/folders/1gAZmwtY3C2bbZCahHcfz1n6obDfkkoFu; https://drive.google.com/drive/folders/1gAZmwtY3C2bbZCahHcfz1n6obDfkkoFu.

Supplementary content related to this article has been published online at https://doi.org/10.1016/j.heliyon.2020.e04876.

## Appendix A Supplementary data

Danbury 2019 - File List Danbury 2019 - File List

Danbury 2019 - outliers Danbury 2019 - outliers

Hartford 2016 - File List Hartford 2016 - File List

Hartford 2016 - outliers Hartford 2016 - outliers

Hartford 2019 - File List Hartford 2019 - File List

Hartford 2019 - outliers Hartford 2019 - outliers

New London 2019 - File List New London 2019 - File List

New London 2019 - outliers New London 2019 - outliers

R Code R Code
