# Supplementary material for: An enhanced procedure for urban mobile methane leak detection
Source: Heliyon. 2020 Oct 9;6(10):e04876. doi: 10.1016/j.heliyon.2020.e04876 (PMC7560587; doi:10.1016/j.heliyon.2020.e04876)
Supplement: R Code [file mmc9.docx]

#==================================================================================

# Author: Tim Kerry Keyes, PhD, Evergreen Business Analytics, LLC

# Code is used to analyze Methane data measurements for “An Enhanced Procedure for Urban Mobile Methane Leak Detection”

# Used by permission only

# Hartford, 2016: https://drive.google.com/open?id=17KOqmlky6Tx_aIOlYi0v2TBRBuZFepkz

# Hartford, 2019: https://drive.google.com/open?id=18oCTzG0Jp8jTDP9y8btJvAFbJvDLvAMh

# Danbury, New London - 2019: https://drive.google.com/open?id=1gAZmwtY3C2bbZCahHcfz1n6obDfkkoFu

#==================================================================================

#CHECK / SET THE R WORKING DIRECTORY

getwd()

#setwd("C:/Users/timke/Documents/Professional/Misc Research Materials/Hazardous Liquid Accidents/Methane Surveys/Hartford - 2016/Baseline Data 2016")

#setwd("C:/Users/timke/Documents/Professional/Misc Research Materials/Hazardous Liquid Accidents/Methane Surveys/Hartford - 2019")

setwd("C:/Users/timke/Documents/Professional/Misc Research Materials/Hazardous Liquid Accidents/Methane Surveys/Danbury Essex New London")

# READ IN FILE LIST

myfiles<-as.data.frame(read.csv("File List.csv", header=TRUE, sep=","))

#myfiles<-subset(myfiles,myfiles$Comment=="Hartford")

#myfiles<-subset(myfiles,myfiles$Comment=="Danbury")

myfiles<-subset(myfiles,myfiles$Comment=="New London")

#myfiles$nleaks<-rep(0,nrow(myfiles))

nrow(myfiles)

tau<-1.645

pi<-3.14159

R<-6371

# READ IN FIRST FILE IN LIST, THEN START "STACKING"; PRODUCE FILE OF BASIC STATS

mydat<-as.data.frame(read.delim(file=as.character(myfiles[1,3]), header = TRUE, sep=""))

mydat$filename<-as.character(myfiles[1,3])

if (nrow(myfiles)>1) {

for (i in 2:nrow(myfiles)) {

# for (i in 2:10) {

if (i==2) {

toprnt<-as.data.frame(cbind(as.character(myfiles[1,3]),nrow(mydat), mean(mydat$CH4), sd(mydat$CH4), mean(mydat$CH4)-tau*sd(mydat$CH4),mean(mydat$CH4)+tau*sd(mydat$CH4)))

}

mydat2<-as.data.frame(read.delim(file=as.character(myfiles[i,3]), header=TRUE, sep=""))

mydat2$filename<-as.character(myfiles[i,3])

mydat<-rbind(mydat,mydat2)

toprnt2<-as.data.frame(cbind(mydat2$filename[i],nrow(mydat2), mean(mydat2$CH4), sd(mydat2$CH4), mean(mydat2$CH4)-tau*sd(mydat2$CH4),mean(mydat2$CH4)+tau*sd(mydat2$CH4)))

toprnt<-rbind(toprnt,toprnt2)

}

write.csv(toprnt,file="File_stats.csv")

}

# OVERALL BASIC STATS

#summary(mydat$CH4)

#hist(mydat$CH4)

#mu<-mean(mydat$CH4)

#sig<-sd(mydat$CH4)

# START PROCESSING EACH FILE HERE

for (l in 1:nrow(myfiles)) {

mydat<-as.data.frame(read.delim(file=as.character(myfiles[l,3]), header = TRUE, sep=""))

mydat$filename<-as.character(myfiles[l,3])

# FIND TOTAL DISTANCE (MILES) COVERED

# SORT THE FILE BY TIMESTAMP

# THEN ACCUMULATE DISTANCE BETWEEN SUCCESSIVE LAT/LONGS USING HAVERSINE

# BE MINDFUL OF BIG STEPS, AS THEY ARE ASSOCIATED WITH START/STOPS OF SENSOR

library("geosphere", lib.loc="~/R/win-library/3.5")

mydat <- mydat[order(mydat$GPS_TIME),]

# THIS IS COMPUTATIONALLY INTENSIVE; DISTHAVERSINE IF USED DEFAULTS TO METERS (1 Mi = 1609.34 Meters)

myfiles$sumdist[l]<-0

mydat$ddiff<-rep(0, nrow(mydat))

for (i in 1:nrow(mydat)) {

mydat$lat[i]=as.double(mydat$GPS_ABS_LAT[i])

mydat$lon[i]=as.double(mydat$GPS_ABS_LONG[i])

if (i>1) {

mydat$ddiff[i]<-distm(c(mydat$lon[i],mydat$lat[i]),c(mydat$lon[i-1],mydat$lat[i-1]),fun=distHaversine)/1609.34

# mydat$ddiff[i]<-distHaversine(c(mydat$lon[i],mydat$lat[i]),c(mydat$lon[i-1],mydat$lat[i-1]))/1609.34

# mydat$ddiff[i]<-sqrt((mydat$lon[i]-mydat$lon[i-1])^2+(mydat$lat[i]-mydat$lat[i-1])^2)*pi/180*R/1.60934

if (mydat$ddiff[i]>0.1) {mydat$ddiff[i]<-0}

myfiles$sumdist[l]=myfiles$sumdist[l]+mydat$ddiff[i]

}

}

# DETECT OUTLIERS

# References:

# https://www.statisticshowto.datasciencecentral.com/modified-thompson-tau-test/

# https://www.mne.psu.edu/cimbala/me345/Lectures/Outliers.pdf

# https://en.wikipedia.org/wiki/Chebyshev%27s_inequality

mydat$outlier<-rep(0, nrow(mydat))

mydat$zscore<-rep(0,nrow(mydat))

mu<-mean(mydat$CH4[mydat$outlier==0],na.rm=TRUE)

sig<-sd(mydat$CH4[mydat$outlier==0],na.rm=TRUE)

n<-nrow(mydat[mydat$outlier==0,])

rr<-tau*(n-1)/(n^0.5*(n-2 + tau^2)^0.5)

k<-3.15

# POSSIBLE OUTLIER COUNT

# nrow(mydat[mydat$CH4>mu+tau*sig,])

# nrow(mydat[mydat$CH4>mu+k*sig,])

# REFINE USING THOMPSON'S TAU METHOD (mu, sig, n, and rr should be updated upon outlier ID)

# mydat <- mydat[order(mydat$CH4, decreasing=TRUE),]

mydat <- mydat[order(mydat$GPS_TIME),]

for (i in 1:nrow(mydat)) {

G<-mu+rr*sig

x1<-mydat$CH4[i]

if (i>1) {x2<-mydat$CH4[i-1]} else {x2<-0}

if (i<=(nrow(mydat)-1)) {x3<-mydat$CH4[i+1]} else {x3<-0}

# if (x1>G) {

if ((x1>G) && (x1>x2) && (x1>x3)) {

mydat$outlier[i]<-1

# mu<-mean(mydat$CH4[mydat$outlier==0],na.rm=TRUE)

# sig<-sd(mydat$CH4[mydat$outlier==0],na.rm=TRUE)

# n<-nrow(mydat[mydat$outlier==0,])

# rr<-tau*(n-1)/(n^0.5*(n-2 + tau^2)^0.5)

# SIZE of OUTLIER RELATIVE TO MEAN CALCULATED HERE

mydat$zscore[i]<-(x1-mu)/sig

}

}

# mu+k*sig

# quantile(mydat$CH4, probs = c(0.99, 0.97, 0.95, 0.90),na.rm=TRUE)

# REVISED OUTLIER COUNT AND CHECK:

# print(nrow(mydat[mydat$outlier==1,]))

# print(nrow(mydat[mydat$CH4>mu+k*sig && mydat$outlier==0,]))

# summary(mydat$CH4[mydat$outlier==1])

# summary(mydat$CH4[mydat$outlier==0])

# DIAGNOSTIC PLOTS

par(mfrow=c(3,2))

hist(mydat$CH4, main="histogram of CH4")

qqnorm(mydat$CH4, main = "Normal Q-Q Plot - All Data",

xlab = "Theoretical Quantiles", ylab = "Sample Quantiles",

plot.it = TRUE, datax = FALSE)

qqline(mydat$CH4, datax = FALSE, distribution = qnorm,

probs = c(0.25, 0.75), qtype = 7)

hist(mydat$CH4[mydat$outlier==0], main="histogram of all non-outliers")

qqnorm(mydat$CH4[mydat$outlier==0], main = "Normal Q-Q Plot - No Outliers",

xlab = "Theoretical Quantiles", ylab = "Sample Quantiles",

plot.it = TRUE, datax = FALSE)

qqline(mydat$CH4[mydat$outlier==0], datax = FALSE, distribution = qnorm,

probs = c(0.25, 0.75), qtype = 7)

# CREATE "SPATIAL WINDOWS" AROUND THE LARGEST LEAK IN AN AREA:

# Start with highest remaining leak ("peak leak").

# Sort other leak data, excluding peak leak, by lat/long.

# Calculate haversine distances from peak leak for ordered (by distance) leaks until 30-meter threshold is met,

# labeling each neighborhood leak as "omit", then stop.

# Step to next non-omitted leak, determined by a sort by size (i.e., move to the next remaining "peak leak").

# Repeat procedure until all TT outliers have been processed (i.e., either remain as a peak leak or were

# omitted as being too close).

# SORT LIST BY DESCENDING CH4, THEN NEED ONLY LOOK AHEAD IN LIST FOR LEAKS IN RADIUS

myleak <- mydat[mydat$outlier==1,]

myleak <- myleak[order(myleak$CH4, decreasing=TRUE),]

thresh<-30

myleak$omit<-rep(0, nrow(myleak))

if (nrow(myleak)>0) {

for (i in 1:nrow(myleak)) {

if (myleak$omit[i]==0) {

j<-i+1

while (j<=nrow(myleak)) {

dH<-1000

if (myleak$omit[j]==0) {

dH<-sqrt((myleak$lat[i]-myleak$lat[j])^2+(myleak$lon[i]-myleak$lon[j])^2)*pi/180*R*1000

if ((dH <= thresh) && (myleak$CH4[i]>myleak$CH4[j])) {myleak$omit[j]<-1}

}

j<-j+1

}

}

}

}

myfiles$nleaks[l]<-nrow(myleak[myleak$omit==0,])

# print(nrow(myleak))

# print(myfiles$nleaks[l])

# print(myfiles$sumdist[l])

# print(myfiles$nleaks[l]/myfiles$sumdist[l])

# MORE DIAGNOSTICS

# hist(mydat$CH4[mydat$outlier==0],main="histogram of all non-outliers")

if (nrow(myleak)>0) {hist(myleak$CH4[myleak$omit==0],main="histogram of unique outliers")}

# summary(myleak$CH4[myleak$omit==0])

# summary(mydat$CH4[mydat$outlier==0])

# head(myleak[order(myleak$GPS_ABS_LAT),],10)

# STACK UP OUTLIER FILES

outfile<-myleak[myleak$omit==0,]

if (l==1) {allout<-outfile}

else if (l>1) {allout<-rbind(allout,outfile)}

# STACK UP ALL FILES

if (l==1) {alldat<-mydat}

else if (l>1) {alldat<-rbind(alldat,mydat)}

# DIAGNOSTIC CHECKING

mydat$dtmp<-rep(0,nrow(mydat))

tmplat<-as.double(41.79460381)

tmplon<-as.double(-72.70206557)

for (i in 1:nrow(mydat)) {

mydat$dtmp[i]<-sqrt((mydat$lat[2211]-mydat$lat[2222])^2+(mydat$lon[2211]-mydat$lon[2222])^2)*pi/180*R*1000

}

}

# END FILE PROCESSING HERE

sum(myfiles$nleaks)

sum(myfiles$sumdist)

nrow(alldat)

# NEED TO DE-DUPE AGAIN - THE ALLOUT FILE

# SORT LIST BY DESCENDING CH4, THEN NEED ONLY LOOK AHEAD IN LIST FOR LEAKS IN RADIUS

allout <- allout[order(allout$CH4, decreasing=TRUE),]

thresh<-30

allout$omitf<-rep(0, nrow(allout))

ptm<-proc.time()

if (nrow(allout)>0) {

for (i in 1:nrow(allout)) {

if (allout$omitf[i]==0) {

j<-i+1

while (j<=nrow(allout)) {

dH<-1000

if (allout$omitf[j]==0) {

dH<-sqrt((allout$lat[i]-allout$lat[j])^2+(allout$lon[i]-allout$lon[j])^2)*pi/180*R*1000

if ((dH <= thresh) && (allout$CH4[i]>allout$CH4[j])) {allout$omitf[j]<-1}

}

j<-j+1

}

}

}

}

proc.time()-ptm

nrow(allout)

nrow(allout[allout$omitf==0,])

summary(allout$CH4[allout$omitf==0])

summary(allout$zscore[allout$omitf==0])

par(mfrow=c(1,1))

#hist(allout$zscore[allout$omitf==0], main="Histogram of Z-score for Predicted Leaks",

# xlab="Standard Units",col="blue",xlim=c(0,40),breaks=c(0,5,10,15,20,25,30,35,40),freq=TRUE)

#main="Histogram of Z-score for Predicted Leaks"

#myhist <- hist(allout$zscore[allout$omitf==0], breaks=c(0,5,10,15,20,25,30,35,40), plot=TRUE, col="blue")

myhist <- hist(allout$zscore[allout$omitf==0], breaks=c(0,5,10,15,20,25,30,35,40), plot=FALSE)

# IF PLOTTING DIRECTLY, NO LOG SCALE...

plot(myhist$breaks[myhist$breaks>0], myhist$counts, type='h',lwd=50, lend=9, col="blue",

ylab="counts",xlab="Standard Units",frame.plot=F)

# IF USING LOG10 SCALE...

myhist$counts<-log10(myhist$counts)

plot(myhist$breaks[myhist$breaks>0], myhist$counts, log="y", type='h',lwd=50, lend=9, col="blue",

ylab="log10 of counts",xlab="Standard Units",frame.plot=F)

outfile<-allout[allout$omitf==0,]

fn<-paste("outliers.csv")

write.csv(outfile,file=fn)

# MAP ALL LEAKS ON A SINGLE MAP

# Load the relevant libraries - do this EVERY time

library(lubridate)

library(ggplot2)

library(dplyr)

library(data.table)

library(ggrepel)

library(tidyverse)

library("ggmap")

#Set API Key

#ggmap::register_google(key = "AIzaSyB06ygVxINSrBxZNbL8mxLEAlo3vySf9Ho")

ggmap::register_google(key = "AIzaSyDRgp9yWL2j9hJRbhNUKUxs2ll1KCFYVeY")

mapdat1<-allout[allout$omitf==0,]

mapdat2<-alldat

mylat<-mean(mapdat2$lat)

mylon<-mean(mapdat2$lon)

# FOR DRILL-DOWN on SPECIAL CASES

#mylat<-mean(41.352)

#mylon<-mean(-72.105)

mapgilbert <- get_map(location = c(lon = mylon, lat = mylat), zoom = 15,

maptype = "roadmap", scale = "auto", res=300)

#mapgilbert <- get_map(location = c(lon = mylon, lat = mylat), zoom = 15,

# maptype = "roadmap", scale = 1)

# BELOW PLOTS CONSTANT SIZE POINTS

# PLOT BOTH OUTLIERS (RED) AND OTHER POINTS (BLUE)

#title="Methane Leaks"

ggmap(mapgilbert) +

# geom_point(data = mapdat1, aes(x = mylon, y = mylat), size = 2, shape = 21, color="black", fill="black")+

geom_point(data = mapdat2, aes(x = lon, y = lat), size = 0.5, shape = 21, color="blue", fill=NA)+

geom_point(data = mapdat1, aes(x = lon, y = lat), size = 1, shape = 21, color="red", fill="red")+

labs(x = "Longitude", y = "Latitude")+

theme(plot.title = element_text(hjust = 0.5))+scale_shape(solid = FALSE)
