# Supplementary material for: An enhanced procedure for urban mobile methane leak detection
Source: Heliyon. 2020 Oct 9;6(10):e04876. doi: 10.1016/j.heliyon.2020.e04876 (PMC7560587; doi:10.1016/j.heliyon.2020.e04876)
Supplement: Appendix [file mmc10.docx]

**An Enhanced Procedure for Urban Mobile Methane Leak Detection**

*25 January, 2020*

Tim Keyes PhD (1)*, Gale Ridge PhD (2), Martha Klein RN MPH (3), Nathan Phillips PhD (4), Robert Ackley (5), Yufeng Yang (4,6)

1. Evergreen Business Analytics, LLC; * corresponding author (Tim.Keyes@EGBANA.com)
2. Steering Committee, 350 CT
3. Sierra Club Connecticut
4. Boston University, Department of Earth and Environment
5. Gas Safety, Inc.
6. Institut National des Sciences Appliquées (INSA), Lyon, France

# APPENDIX

## High-level Steps for File and Data Processing

1. Upload files (.DAT, .KML) created through the survey process to a shared Google Drive.
2. View .KML files in Google Earth Pro and determine which individual files are associated with a town’s survey, versus files that represent the vehicle being “in transit” to or from the vehicle’s starting and ending point for the day of the survey (readings are taken whenever the vehicle is in use). The latter files are excluded from analysis.
3. Create a survey “File List” for use in subsequent outlier detection and de-duplication (files associated with a micro-area within a town).
4. For each file in the File List,
   1. Record the number of observations (individual CH_4_ measurements taken).
   2. Perform the modified Thompson’s Tau outlier detection methodology; an outlier as determined by this methodology is an initial indication of a predicted leak.
   3. Perform de-duplication methodology to omit repeated observations of the same predicted leak within a threshold radius (30 meters as previously described), keeping the largest outliers as final indications of predicted leaks *for the file*.
5. Once all individual files in the File List are processed,
   1. Aggregate each file’s final predicted leaks into a combined, town-level data set.
   2. Repeat the de-duplication methodology in step 4) a. above, for the aggregated list of predicted leaks, *across files*. This produces the final predicted leaks associated with a town’s survey.
6. Plot GPS points associated with each survey measurement, superimposed with GPS points associated with each predicted leak.

## Supplementary Files Accompanying this Paper

R Programming Code: R Code.docx

## Input Files, Control Lists and Output Files for Each Survey

| **Survey Location** | **Location of Input Files** | **List of .Dat Files Processed** | **Output Files (Predicted Leaks)** |
| --- | --- | --- | --- |
| Hartford, 2016 | [Google Drive](https://drive.google.com/open?id=17KOqmlky6Tx_aIOlYi0v2TBRBuZFepkz) | Danbury 2016 - File List.csv | Danbury 2016 - outliers.csv |
| Hartford, 2019 | [Google Drive](https://drive.google.com/open?id=18oCTzG0Jp8jTDP9y8btJvAFbJvDLvAMh) | Hartford 2019 - File List.csv | Hartford 2019 - outliers.csv |
| Danbury, 2019 | [Google Drive](https://drive.google.com/open?id=1gAZmwtY3C2bbZCahHcfz1n6obDfkkoFu) | Danbury 2019 - File List.csv | Danbury 2019 - outliers.csv |
| New London, 2019 | [Google Drive](https://drive.google.com/open?id=1gAZmwtY3C2bbZCahHcfz1n6obDfkkoFu) | New London 2019 - File List.csv | New London - outliers.csv |
